# Upregulation of GLS1 Isoforms KGA and GAC Facilitates Mitochondrial Metabolism and Cell Proliferation in Epstein–Barr Virus Infected Cells

**DOI:** 10.3390/v12080811

**Published:** 2020-07-27

**Authors:** Gayathri Krishna, Vinod Soman Pillai, Mohanan Valiya Veettil

**Affiliations:** Virology Laboratory, Department of Biotechnology, Cochin University of Science and Technology, Cochin 682022, Kerala, India; gayathriradhan@gmail.com (G.K.); vinodsoman08@gmail.com (V.S.P.)

**Keywords:** EBV, KGA, GAC, glutaminolysis, mitochondrial metabolism, cell proliferation

## Abstract

Epstein–Barr virus or human herpesvirus 4 (EBV/HHV-4) is a ubiquitous human virus associated with a wide range of malignant neoplasms. The interaction between EBV latent proteins and host cellular molecules often leads to oncogenic transformation, promoting the development of EBV-associated cancers. The present study identifies a functional role of GLS1 isoforms KGA and GAC in regulating mitochondrial energy metabolism to promote EBV-infected cell proliferation. Our data demonstrate increased expression of GLS1 isoforms KGA and GAC with mitochondrial localization in latently EBV-infected cells and de novo EBV-infected PBMCs. c-Myc upregulates KGA and GAC protein levels, which in turn elevate the levels of intracellular glutamate. Further analysis demonstrated upregulated expression of mitochondrial GLUD1 and GLUD2, with a subsequent increase in alpha-ketoglutarate levels that may mark the activation of glutaminolysis. Cell proliferation and viability of latently EBV-infected cells were notably inhibited by KGA/GAC, as well as GLUD1 inhibitors. Taken together, our results suggest that c-Myc-dependent regulation of KGA and GAC enhances mitochondrial functions to support the rapid proliferation of the EBV-infected cells, and these metabolic processes could be therapeutically exploited by targeting KGA/GAC and GLUD1 to prevent EBV-associated cancers.

## 1. Introduction

Epstein–Barr virus (EBV) or human herpesvirus 4 (HHV-4) is an oncogenic virus that infects and establishes latency in more than 90% of the world’s human population. EBV is associated with a variety of B-cell lymphomas, such as Burkitt’s lymphoma, Hodgkin’s lymphoma, and post-transplantation lymphoproliferative disorders, and two epithelial cancers, nasopharyngeal carcinoma and gastric carcinoma [1,2,3]. Depending on the expression of EBV-latent proteins, distinct latency patterns are associated with specific EBV-associated cancers. Burkitt’s lymphoma is associated with the expression of type I latency genes, EBV-nuclear antigen 1 (EBNA1) and EBV-encoded RNAs (EBERs). Hodgkin’s lymphoma and nasopharyngeal carcinoma are characterized by the expression of type II latency genes, EBNA1, latent membrane protein 1 (LMP1), LMP2A, LMP2B, and EBERs. In post-transplantation lymphoproliferative diseases and in vitro immortalized lymphoblastoid cell lines (LCLs), EBV has the most composite expression profile, latency III, which involves the expression of five EBNAs (EBNA1, 2, 3A, 3B, 3C), two LMPs (LMP1, LMP2A), and two EBERs [4,5,6].

Although the pattern of latent gene expression varies among EBV-associated cancers, they share several cellular metabolic adaptations that influence the uptake of nutrients to support rapid cell proliferation, cell growth, and survival [7]. c-Myc is an important cellular oncogene which co-ordinates viral gene expression and metabolic reprograming in EBV-associated cancers [8]. Notably, in Burkitt’s lymphoma, chromosomal translocation of c-Myc to the IgG locus leads to overexpression of c-Myc, resulting in increased cell proliferation and malignant transformation [9]. c-Myc upregulation has also been reported in about 90% of EBV-associated nasopharyngeal carcinomas [10,11,12]. Additionally, the activation of c-Myc transcription by EBNA2 was reported to play an instrumental role in EBV-infected LCL proliferation and survival [13]. c-Myc is a transcription factor which regulates the expression of many genes associated with the cellular metabolic processes to meet the high metabolic and bioenergetics demands of cancer cells [14].

Aerobic glycolysis and glutaminolysis are the major metabolic pathways used by cancer cells to fuel their bioenergetic and biosynthetic needs [15]. As the energy derived from aerobic glycolysis is not sufficient to meet the energy requirements of actively dividing cancer cells, they also depend on increased glutamine uptake and glutaminolysis to sustain a functional TCA cycle for the production of energy, reductive equivalents, and the biosynthesis of various macromolecules supporting tumor growth and proliferation [16]. Glutaminolysis involves the deamination of glutamine to glutamate catalyzed by GLS. Glutamate is subsequently converted to a TCA cycle intermediate, alpha-ketoglutarate, mediated by glutamate dehydrogenases or aminotransferases that replenish the TCA cycle.

The mitochondrial enzyme GLS1 plays a crucial role in maintaining metabolism and homeostasis. In mammalian cells, GLS1 encodes two isoforms: kidney (K-type) glutaminase (KGA) and glutaminase C (GAC) [17]. Despite distinct tissue distribution patterns, the functionality of both isoforms remains the same. GAC is found to be the more catalytically active and predominant isoform, with implications in tumor metabolism [18]. However, the involvement of KGA and GAC in oncogenic cellular energy metabolism and cell proliferation, as well as its connection with the aberrantly expressed c-Myc, is not completely understood in EBV-associated cancers. We hypothesized that the expression of KGA and GAC may be an adaptation of rapidly proliferating EBV-infected cells to upgrade the efficiency of glutaminolysis for the sustenance of the increased energy demands of tumor metabolism. Thus, understanding the interplay of KGA and GAC isoforms in the oncogenic cellular energy metabolism of EBV-infected cancers could unravel the bioenergetics in EBV’s pathobiology.

In the present study, we demonstrate that EBV infection upregulates the expression of GLS1 isoforms KGA and GAC, which in turn are regulated by c-Myc. Both KGA and GAC localize to the mitochondria, where they are involved in the conversion of glutamine into glutamate and the formation of alpha-ketoglutarate, which is a critical molecule in mitochondrial energy metabolism. Our data indicate that both KGA and GAC isoforms are key determinants of mitochondrial metabolism and the rapid proliferation of latently EBV-infected cells. These findings suggest that targeting glutaminolysis by inhibiting KGA and GAC isoforms could result in the development of an innovative therapeutic strategy for limiting EBV-associated cancers.

## 2. Materials and Methods 

### 2.1. Cells

EBV-positive Burkitt’s lymphoma cell lines Raji, Namalwa, Jiyoye, EBV-producing marmoset cell line B95-8, nasal epithelial carcinoma cell line RPMI 2650, and HEK 293T cell line were obtained from the National Centre for Cell Science (NCCS), Pune, India. EBV-positive nasopharyngeal cell line C666-1 and EBV-negative lymphoma cell line BJAB were a gift from Dr. Bala Chandran (University of South Florida, Tampa, FL, USA). Peripheral blood mononuclear cells (PBMC) were purchased from HiMedia laboratories, India. The EBV-transformed lymphoblastoid cell line (LCL) was generated in our laboratory by infection of PBMC with EBV using standard protocols [19]. BJAB, Raji, Namalwa, C666-1, Jiyoye, B95-8, LCL, and PBMCs were cultured in RPMI-1640 (Gibco BRL, Grand Island, NY, USA) supplemented with 10% fetal bovine serum (FBS) and 1% penicillin-streptomycin. HEK 293T cells were maintained in DMEM (Gibco BRL, Grand Island, NY, USA) supplemented with 10% FBS and 1% penicillin-streptomycin. RPMI 2650 cells were cultured in DMEM supplemented with 1 mM sodium pyruvate, 20% FBS, and 1% penicillin-streptomycin. All cell lines were maintained at 37 °C with 5% CO_2_.

### 2.2. Virus

The EBV lytic cycle was induced with Tetradecanoyl phorbol 12-myristate 13-acetate (TPA) in B95-8 cells. Briefly, B95-8 cells treated with 20 ng/mL TPA were incubated at 37 °C for 5 days. The spent media was centrifuged at 5000 rpm for 5 min at 4 °C. The supernatant was filtered and ultracentrifuged at 70,000 rpm for 2 h at 4 °C. The viral pellets were dissolved in PBS [19]. Viral DNA was isolated and real-time PCR was performed to determine the virus titer by comparison with a standard curve prepared from Namalwa cell DNA [19]. 

### 2.3. Antibodies and Reagents

TPA, polybrene, polyethylenimine (PEI), bis-2-(5-phenylacetamido-1,3,4-thiadiazol-2-yl) ethyl sulfide (BPTES), ebselen, and 10074-G5 were obtained from Sigma (St. Louis, MO, USA). The antibodies used in this study are listed in the following Table 1. 

### 2.4. Lentivirus Production

Lentiviruses encoding c-Myc shRNA and control shRNA were produced by transiently transfecting HEK 293T cells with lentiviral packaging plasmids (Gag-Pol, Rev and VSV-G) and plasmid encoding c-Myc shRNA (Cat #29435, Addgene, Cambridge, MA, USA) or control shRNA, as described previously [20]. The supernatants containing lentiviruses were collected and filtered after 72 h. Cells were transduced with c-Myc lentivirus or control shRNA in the presence of polybrene (5 µg/mL). Transduction efficiency was estimated by Western blot.

### 2.5. Glutamate Assay

Intracellular glutamate concentrations were determined using the glutamate calorimetric assay kit (Biovision, Milpitas, CA, USA). Equal numbers of cells were seeded for all experiments. Briefly, cell pellets were collected at different time points by centrifugation at 5000 rpm for 5 min. The cell pellets were homogenized using the assay buffer and centrifuged at 13,000 rpm for 10 min to collect the lysates. Cell lysates were assayed for glutamate levels using the glutamate calorimetric assay kit, as per the manufacturer’s protocol. The absorbance was measured at 450 nm with iMark Microplate absorbance reader (Biorad, Hercules, CA, USA). 

### 2.6. Alpha-Ketoglutarate Assay

Alpha-ketoglutarate calorimetric assay kit (Biovision, Milpitas, CA, USA) was used to measure the intracellular alpha-ketoglutarate concentrations. Equal numbers of cells were seeded for all experiments. Briefly, cell pellets collected at different time points by centrifugation at 5000 rpm for 5 min were homogenized using the assay buffer and centrifuged at 13,000 rpm for 10 min to collect the lysates. Cell lysates were assayed for alpha-ketoglutarate concentration using alpha-ketoglutarate calorimetric assay kit, as per the manufacturer’s protocol. The absorbance was measured at 570 nm with iMark Microplate absorbance reader (Biorad). 

### 2.7. Quantitative Real-Time Reverse-Transcription PCR

Total RNA was extracted using TRI reagent (Sigma, St. Louis, MO, USA) and treated with DNase (Promega, Madison, WI, USA). Total RNA (2 µg) was used for cDNA synthesis using a High Capacity cDNA reverse transcription kit (Applied Biosystems, Foster City, CA, USA). Quantitative real-time PCR was performed using Power SYBR green (Applied Biosystems) and Applied Biosystems 7300 real-time PCR system. GAPDH expression was used for normalizing real-time PCR data. The ΔΔCt method was used to calculate the relative gene expression compared to the control. Primer sets used for real-time PCR amplifications are shown in Table 2:

### 2.8. Western Blot

Cells were lysed in radioimmunoprecipitation assay (RIPA) lysis buffer (25 mM Tris-HCl pH 7.6, 150 mM NaCl, 5 mM EDTA, 1% Triton X-100, 1% sodium deoxycholate, 0.1% SDS, 1 mM PMSF -phenymethyl sulfonyl fluoride) and protease inhibitor cocktail (Sigma). The lysates were centrifuged at 15,000 rpm for 10 min at 4 °C and the supernatants were collected. Protein concentration was determined by the Pierce BCA protein assay kit (Thermo Fisher Scientific, Waltham, MA, USA). Lysates were heated at 95 °C for 5 min with Laemmli sample buffer. Proteins were separated by SDS-PAGE and blotted onto nitrocellulose membrane (Biorad). Membranes were blocked for an hour in 5% skimmed milk and incubated with specific primary antibodies at 4 °C overnight, followed by appropriate horseradish peroxidase (HRP)-conjugated secondary antibodies for 2 h at room temperature (RT). Enhanced chemiluminescence substrate (Pierce, MA, USA) was used to visualize specific bands on a Biorad Chemidoc. The band intensities were measured using ImageJ software and were normalized using β-actin.

### 2.9. Immunofluorescence Assay

Equal numbers of cells were spotted onto 12-well spot slides (Thermo Fisher Scientific) or grown in chamber slides and were fixed with acetone or 4% paraformaldehyde. Cells were permeabilized using 0.2% Triton X-100 and blocked with Image-iT FX Signal enhancer (Invitrogen, Carlsbad, CA, USA) for 20 min and incubated with specific primary antibody at 4 °C overnight and appropriate Alexa 594 or Alexa 488 secondary antibody for 2 h at RT. Mitochondria were stained with 250 nM Mito Tracker Deep Red FM (Invitrogen). Cells were mounted with anti-fade reagent containing DAPI (4′ 6-diamidino 2-phenylindole; Thermo Fisher Scientific) and visualized by Nikon fluorescence microscope.

### 2.10. BrdU Cell Proliferation Assay

The cell proliferation assay was performed using the BrdU cell proliferation assay kit (Cell Signaling Technology). Briefly, equal numbers of cells were plated and treated with vehicle control (DMSO), BPTES (Bis-2-(5-phenylacetamido-1,3,4-thiadiazol-2-yl) ethyl sulfide) (5–20 µM), for 48 h and 72 h. After treatment, the cells were incubated with BrdU for 2 h, followed by incubation with primary and secondary antibodies, as per the manufacturer’s protocol. The absorbance was measured at 450 nm with iMark Microplate absorbance reader (Biorad).

### 2.11. MTT Assay

Equal numbers of cells were seeded and treated with vehicle control (DMSO), BPTES (20 µM), and ebselen (20 µM) for 72 h, after which the cells were incubated with MTT reagent (Thermo Fisher Scientific) for 4 h at 37 °C and solubilized with SDS in HCl at 37 °C for 4 h. The absorbance was measured at 570 nm with iMark Microplate absorbance reader (Biorad).

## 3. Results

### 3.1. GLS1 Isoforms KGA and GAC Are Expressed at High Levels in Latently EBV-Infected Lymphoma Cells

**Figure 1:**
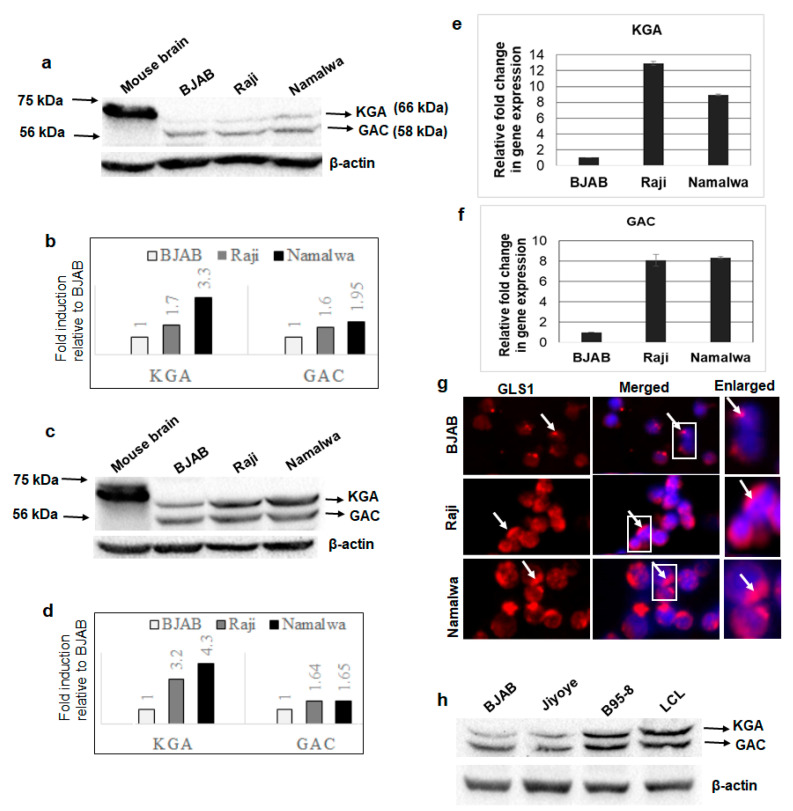
Expression of GLS1 isoforms KGA and GAC in latently EBV-infected B-lymphoma cells. (**a**) Whole cell lysates from EBV-negative cell line BJAB and EBV-positive cell lines Raji and Namalwa were collected, and GLS1 protein levels were determined by Western blot using anti-GLS1 antibody. Mouse brain tissue lysate was used as a positive control. β-actin was used as loading control. (**b**) The intensities of the 66 and 58 kDa bands were quantified separately by ImageJ software and compared with EBV-negative BJAB cells to calculate fold change. (**c**) Whole cell lysates from BJAB, Raji, and Namalwa cells were collected and blotted using KGA/GAC isoform-specific antibody. Mouse brain tissue lysate was used as a positive control. β-actin was used as loading control. (**d**) The band intensities of KGA 66 kDa and GAC 58 kDa were quantified and plotted as fold changes compared to control BJAB cells. (**e**,**f**) RNA isolated from BJAB, Raji, and Namalwa cells were analyzed for KGA (**e**) and GAC (**f**) expression by RT-PCR. Relative levels of gene transcripts were calculated using the ∆∆CT method after normalizing with the expression of GAPDH, and the fold change was compared against the EBV-negative cell line. Error bars represent the mean ± SD of two independent experiments. (**g**) BJAB, Raji, and Namalwa cells were incubated with anti-GLS1 antibody, followed by secondary antibody conjugated to Alexa Flour 594 (red). Nuclei were stained with DAPI (blue). Boxed areas are enlarged on the right panel. Arrows indicate GLS1 staining in the cytoplasm. Magnification ×20. (**h**) Whole cell lysates from EBV-negative cell line BJAB and EBV-positive cell lines Jiyoye, B95-8, and LCL were collected and Western blotted for KGA and GAC expression using KGA/GAC isoform-specific antibody. β-actin was used as loading control.

To examine the expression profile of GLS1 isoforms in EBV-associated cancers, cell lysates from EBV-positive lymphoma cell lines Raji and Namalwa and EBV-negative lymphoma cell line BJAB were Western blotted using a GLS1 specific antibody. Mouse brain tissue extract was used as a positive control. While the positive control detected a single band at 66 kDa, all the lymphoma cells including BJAB detected GLS1 as two distinct bands: one upper band at 66 kDa, which is same as the positive control, and one lower band at 58 kDa (Figure 1a), probably representing the KGA and GAC isoforms of GLS1. Fold change was calculated for each band, and both 66 and 58 kDa band intensities were increased in EBV-positive cells. The 66 kDa KGA and 58 kDa GAC intensity was increased 1.7 and 3.3-fold and 1.6 and 1.95-fold in Raji and Namalwa cells, respectively, compared to the EBV-negative BJAB cells (Figure 1b). To confirm that these two bands are KGA and GAC isoforms, whole cell lysates from BJAB, Raji, and Namalwa cells, and the positive control mouse brain tissue lysates, were Western blotted using another antibody which specifically detects the KGA/GAC isoforms. The Western blot analysis confirmed KGA and GAC isoforms with molecular weights at 66 and 58 kDa (Figure 1c), respectively, as detected by the GLS1 antibody bands in Figure 1a, with higher band intensities in EBV-positive Raji and Namalwa cells than the negative control BJAB cells. The relative fold increase in KGA and GAC protein expression is shown in the graph (Figure 1d). We further determined the mRNA levels of KGA and GAC in both EBV-negative and positive cell lines by RT-PCR. A significant upregulation of KGA and GAC mRNA levels was observed in latently EBV-infected Raji (12 and eight-fold, respectively; Figure 1e,f) and Namalwa cells (8.5 and eight-fold, respectively; Figure 1e,f) compared to BJAB cells. The expression of KGA and GAC was also examined by GLS1 immunofluorescence analysis (IFA). A marked increase in GLS1 staining in the cytoplasm was observed in Raji and Namalwa compared to BJAB cells, which displayed low levels of GLS1 staining (Figure 1g).

We also determined the expression of KGA and GAC in three additional EBV-positive cell lines, Jiyoye, B95-8, and LCL, by Western blot. Compared to EBV-negative BJAB cells, KGA and GAC proteins were highly expressed in B95-8 and LCL, whereas only a slight increase in expression was observed in Jiyoye (Figure 1h). These results confirmed that the isoforms of GLS1- KGA and GAC- are highly expressed in most of the EBV-infected lymphoma cells.

### 3.2. KGA and GAC Isoforms of GLS1 Are Upregulated in EBV-Infected Nasopharyngeal Carcinoma Cells

**Figure 2:**
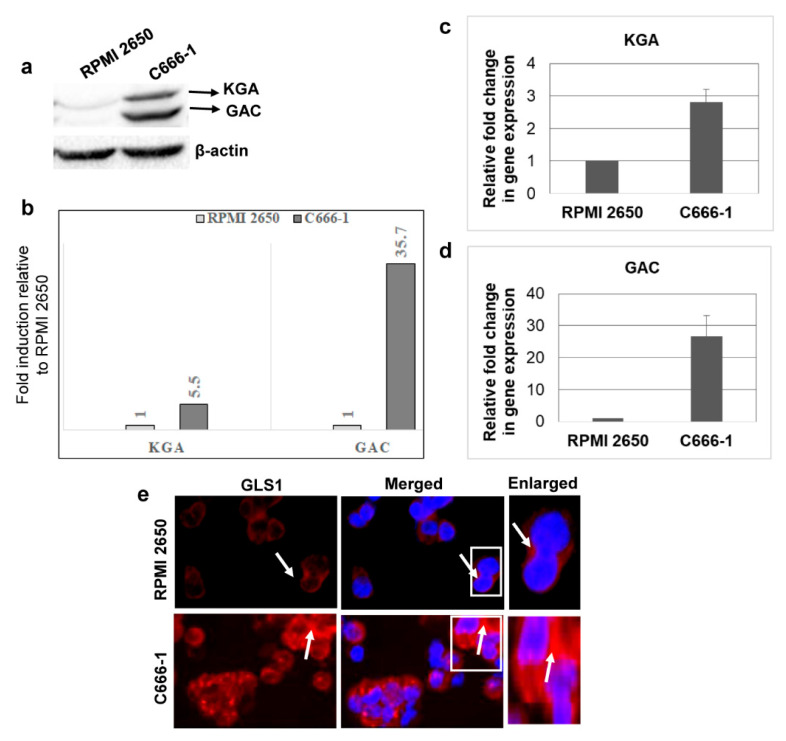
KGA and GAC expression in EBV-infected nasopharyngeal carcinoma cells. (**a**) Whole cell lysates from EBV-negative nasal carcinoma cell line RPMI 2650 and EBV-positive nasopharyngeal carcinoma cell line C666-1 were Western blotted for GLS1 with anti-GLS1 antibody, which can detect both KGA and GAC. β-actin was used as loading control. (**b**) Quantification of Western blot bands. KGA and GAC expression difference between EBV-negative RPMI 2650 and EBV-positive C666-1 cells were quantified as fold changes and plotted. (**c**,**d**) RT-PCR for KGA (**c**) and GAC (**d**) expression in RNA isolated from RPMI 2650 and C666-1 cell lines. GAPDH was used as an internal control. The fold change was calculated compared to the EBV-negative cell line and represented as the mean ± SD of three independent experiments. (**e**) GLS1 localization of RPMI 2650 and C666-1 cells was determined by immunofluorescence analysis with anti-GLS1 antibody, followed by Alexa Flour 594 (red) conjugated secondary antibody. Nuclei were stained with DAPI (blue). Arrow marks indicate cytoplasmic staining of GLS1. Magnification ×20. Right panels show enlarged images of the boxed areas.

We next sought to investigate whether GLS1 isoforms are also upregulated in EBV-associated epithelial cancer cells using EBV-positive nasopharyngeal cancer cell line C666-1 and EBV-negative nasal epithelial carcinoma cell line RPMI 2650. When we evaluated the expression of GLS1 by Western blot, KGA and GAC protein levels were higher, with a 5.5 and 35.7-fold change in EBV-positive C666-1 compared to EBV-negative RPMI 2650 cells (Figure 2a,b). Upregulation of KGA and GAC in EBV-positive C666-1 cells was further confirmed by RT-PCR with 2.5 fold and 25-fold increases in KGA (Figure 2c) and GAC mRNA (Figure 2d) levels, respectively, compared to the EBV-negative RPMI 2650 cells. Additionally, IFA showed increased cytoplasmic GLS1 staining in C666-1 compared to RPMI 2650 cells (Figure 2e). These results demonstrate that the expression of KGA and GAC isoforms of GLS1 was also elevated in latently EBV-infected nasopharyngeal carcinoma cells.

### 3.3. KGA and GAC Localized to the Mitochondria of EBV-Infected Cancer Cells

**Figure 3:**
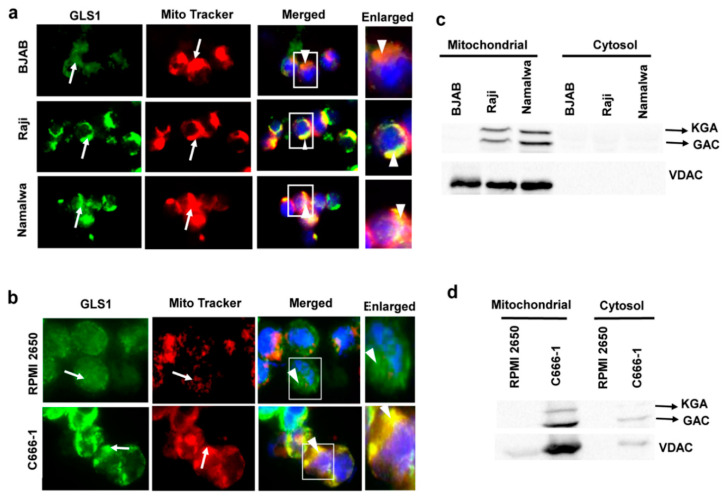
Mitochondrial localization of KGA and GAC in EBV-infected cells. (**a**) BJAB, Raji, and Namalwa and (**b**) RPMI 2650 and C666-1 were fixed, after an incubation of 45 min with MitroTracker (red) at 37 °C. Cells were then washed, fixed, and stained using KGA/GAC isoform-specific antibody, followed by secondary antibody conjugated to Alexa Flour 488 (green). Nuclei were stained with DAPI (blue). Arrows indicate mitochondrial and KGA/GAC staining, and arrow heads indicate their colocalization with the mitochondrial marker. Magnification ×60. Boxed areas are enlarged on the rightmost panel. (**c**,**d**) GLS1 protein levels in mitochondrial fractions of EBV-infected cells. Cell lysates from BJAB, Raji, and Namalwa cell lines (**c**) and RPMI 2650 and C666-1 (**d**) cell lines were fractionated into mitochondrial and cytosolic fractions and Western blotted for GLS1 with anti-GLS1 antibody. The purity of mitochondria was confirmed by VDAC using anti-VDAC antibody.

GLS1 is a mitochondrial enzyme; however, the subcellular localization of KGA and GAC isoforms is debatable in different cell types. Some studies have reported the localization of both KGA and GAC isoforms in mitochondria, while, in a few studies, KGA is exclusively cytoplasmic [18,21]. To determine whether KGA and GAC isoforms are sorted into the mitochondria in latently EBV-infected cells, we pulsed BJAB, Raji, and Namalwa cells with MitoTracker, followed by fixation and staining with GLS1 specific antibody. In the infected Raji and Namalwa cells, KGA/GAC and MitoTracker displayed distinct colocalization, indicating that they are preferentially localized to the mitochondria. In contrast, in the EBV-negative BJAB cells, localization of KGA/GAC into the mitochondria, as well as its colocalization with the MitoTracker, was markedly decreased (Figure 3a). We also determined the translocation of KGA/GAC into the mitochondria by IFA, using MitoTracker and GLS1 antibody in C666-1 and RPMI 2650 cells. Similar results showing strong mitochondrial localization of KGA/GAC were observed in C666-1 cells compared to the control RPMI 2650 cells, which exhibited a clear reduction in mitochondrial KGA/GAC and no significant colocalization with the mitochondrial marker (Figure 3b).

To further confirm the subcellular localization of KGA and GAC isoforms in latently EBV-infected cells, we fractionated the BJAB, Raji, and Namalwa cell lines into mitochondrial and cytosolic fractions and Western blotted with GLS1 antibody. The Western blot analysis showed strong signals for KGA and GAC in the mitochondrial fractions of EBV-positive Raji and Namalwa cells compared with the cytosol fraction, which showed faint bands of KGA and GAC (Figure 3c). The same fractions were Western blotted with VDAC antibody as a mitochondrial marker to verify the purity of mitochondrial fractions (Figure 3c). We next assessed the mitochondrial and cytosol fraction of EBV-positive C666-1 cells for KGA and GAC. EBV-positive C666-1 cell line presented strong bands of KGA and GAC compared to the control cell line RPMI 2650 (Figure 3d). These studies suggest that the upregulation of KGA/GAC in EBV-infected cells is accompanied by a concomitant increase in the level of mitochondrial KGA/GAC.

### 3.4. De Novo EBV Infection Induces GLS1 Expression and Mitochondrial Localization

**Figure 4:**
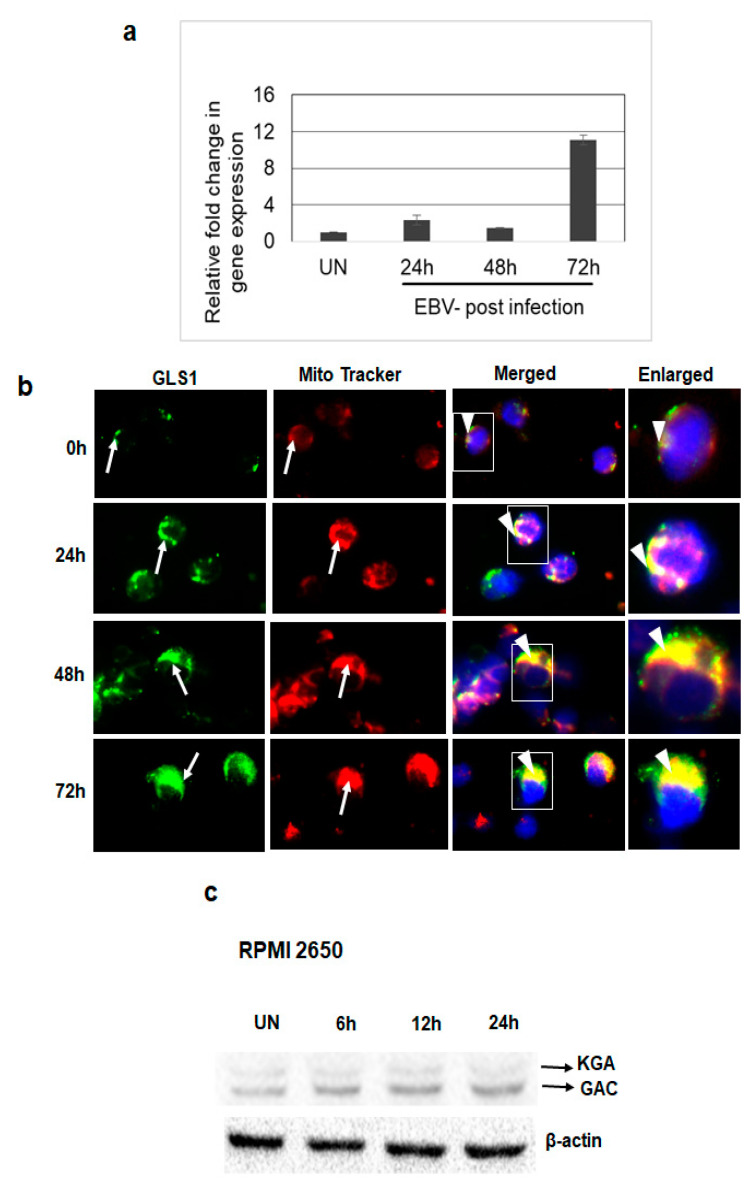
GLS1 expression in EBV-infected PBMCs in vitro. (**a**,**b**) PBMCs were left uninfected or infected with ~20 MOI of EBV for 24 h, 48 h, and 72 h at 37 °C. (**a**) Total RNA was extracted from uninfected and EBV-infected PBMCs and the expression of GLS1 was determined by real-time RT-PCR. GAPDH was used as an internal control. The fold change was calculated and compared to the uninfected control. Error bars represent the mean ± SD of two independent experiments. (**b**) Uninfected or EBV-infected PBMCs were incubated for 45 min with MitroTracker (red) at 37 °C and stained using KGA/GAC isoform-specific antibody, followed by secondary antibody conjugated to Alexa Flour 488 (green). Nuclei were stained with DAPI (blue). Arrows indicate KGA/GAC and mitochondrial staining, and arrow heads indicate their colocalization with the mitochondrial marker. Magnification ×60. Boxed areas are enlarged in the rightmost panel. (**c**) RPMI 2650 cells were left uninfected (UN) or infected with EBV for 6 h, 12 h, and 24 h. Cell lysates were Western blotted using KGA/GAC antibody. β-actin was used as loading control.

Since we observed upregulated expression and mitochondrial localization of KGA and GAC in latently EBV-infected cells, we further investigated the expression and localization of GLS1 during the de novo infection of human PBMCs by EBV. PMBCs left uninfected or infected with EBV for 24 h, 48 h, and 72 h time points, RNA isolated post-infection, and GLS1 mRNA levels were measured by RT-PCR. We observed a time dependent increase in GLS1 expression in PBMCs infected with EBV at different time points, which peaked at 72 h post-infection (Figure 4a).

To evaluate whether KGA and GAC in EBV-infected PBMCs localize to the mitochondria, we analyzed the colocalization of KGA/GAC staining with the MitoTracker in uninfected PBMCs and PBMCs infected with EBV for 24 h, 48 h and 72 h time points. Analysis of immunofluorescence showed that the EBV-infected cells exhibited higher levels of KGA/GAC staining and colocalization with the mitochondrial marker (Figure 4b). The colocalization increased in a time-dependent manner from 24 h to 72 h compared to the uninfected PBMC, suggesting that the expression of KGA and GAC and its mitochondrial localization is associated with EBV infection.

To further investigate whether EBV infection induces GLS1 expression following de novo infection of epithelial cells, EBV-negative nasal epithelial carcinoma cells RPMI 2650 were left uninfected or infected with EBV for different time points. The protein levels of KGA and GAC were determined at different time points post-infection by Western blot. Compared to the uninfected cells, EBV infection increased the expression of KGA and GAC in a time-dependent manner in RPMI 2650 cells (Figure 4c). Collectively, these results suggest that EBV upregulates KGA and GAC expression during de novo infection.

### 3.5. c-Myc Regulates the Expression of KGA and GAC in EBV-Infected Cells

**Figure 5:**
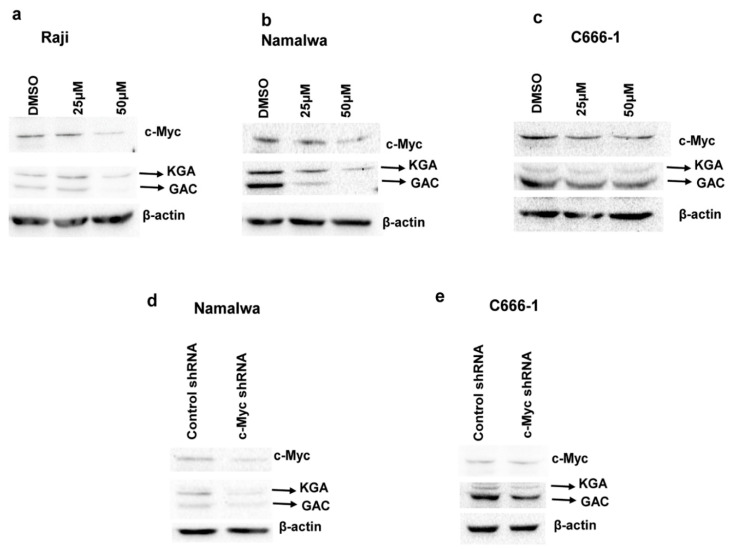
c-Myc regulates KGA and GAC protein expression in EBV-infected cells. (**a**–**c**) Raji (**a**), Namalwa (**b**), and C666-1 (**c**) were treated with the vehicle control DMSO or 25 µM or 50µM of c-Myc inhibitor 10074-G5 for 24 h. Cell lysates collected from the treated cells were Western blotted for c-Myc and GLS1 expression with anti-c-Myc and anti-GLS1 antibodies, respectively. β-actin was used as loading control. Percentages of c-Myc inhibition in the inhibitor treated cells were compared with the vehicle control treated cells. (**d**,**e**) Namalwa (**d**) and C666-1 (**e**) were transduced with lentiviral constructs of control shRNA or c-Myc shRNA for 72 h. Whole cell lysates collected after 72 h of transduction from the control shRNA and c-Myc shRNA cells were used for determining the protein levels of c-Myc, KGA, and GAC by Western blot analysis with anti-c-Myc and anti-GLS1 antibodies, respectively. β-actin was used as loading control. Percentage inhibition of c-Myc was calculated and compared with the control shRNA cells.

c-Myc has been shown to regulate glutaminolysis through the translational activation of GLS1 [22], and GLS1 expression is increased in c-Myc-dependent cancers [22,23]. Regardless of the presence of a c-Myc binding site on the glutaminase gene, the mRNA levels of glutaminase do not change in response to alterations in c-Myc and the regulation is at glutaminase protein levels [22]. To address whether c-Myc plays any role in regulating the expression of KGA and GAC in EBV-infected cells, we first treated EBV-positive Raji, Namalwa, and C666-1 cells with the c-Myc inhibitor 10074-G5, which inhibits the transcriptional activity of c-Myc by disrupting Myc-Max heterodimerization [24]. Immunoblot analysis after 24-h treatment showed a dose-dependent reduction in KGA and GAC expression in Raji (Figure 5a), Namalwa (Figure 5b), and C666-1 (Figure 5c) when treated with 25 µM and 50 µM of 10074-G5 compared to their respective DMSO controls.

To confirm the role of c-Myc in the regulation of KGA and GAC protein levels in EBV-infected cells, we transfected EBV-positive Namalwa and C666-1 cells with lentiviruses encoding c-Myc shRNA or with scrambled shRNA as a negative control. Knockdown resulted in a 50%–60% decrease in c-Myc expression, as analyzed by Western blot, in Namalwa (Figure 5d) and C666-1 (Figure 5e) cell lines.

KGA and GAC expression, as shown in Figure 5d,e, was markedly decreased in c-Myc shRNA transduced cells compared to control shRNA cells. Collectively, these data indicate that, in EBV-infected cells, c-Myc plays a crucial role in regulating the expression of KGA and GAC.

### 3.6. KGA and GAC Expression Increases Intracellular Glutamate Levels in EBV-Infected Cells

**Figure 6:**
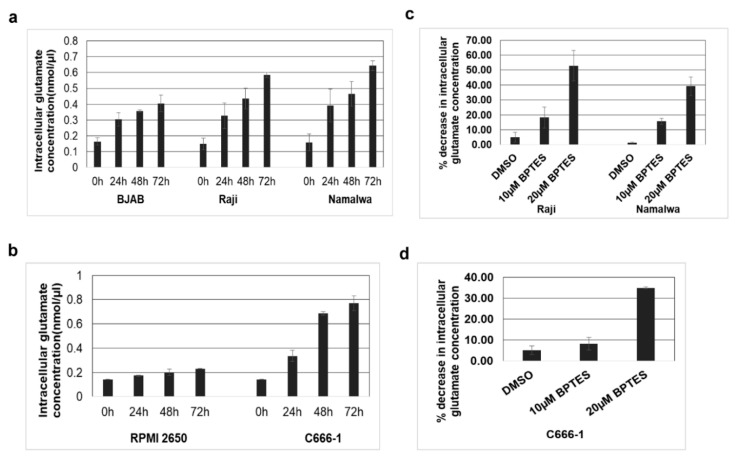
KGA and GAC expression increase intracellular glutamate levels. (**a**) EBV-negative cell line BJAB and EBV-positive cell lines Raji and Namalwa were cultured and intracellular glutamate concentration was determined in the cell lysates collected at various time points using glutamate assay kit. (**b**) Intracellular glutamate concentration in the cell lysates collected at various time points from EBV-negative cell line RPMI 2650, and EBV-positive cell line C666-1. Glutamate concentration is expressed in nanomoles per microliter. (**c**,**d**) Namalwa, Raji, and C666-1 cell lines were treated with control DMSO or with two different concentrations of BPTES 10 µM and 20 µM for 72 h. Intracellular glutamate concentration was determined in the lysates collected after 72 h. Graphs represent the percentage decrease in intracellular glutamate concentration in Raji, Namalwa (**c**) C666-1 (**d**) Error bars represent the mean ± SD of two independent experiments.

We next evaluated the impact of upregulated expression of KGA and GAC in the conversion of glutamine to glutamate in the mitochondria. To ascertain this, intracellular glutamate levels in EBV-positive Raji, Namalwa, and C666-1 as well as in the EBV-negative control BJAB and RPMI 2650 cells were measured. As shown in Figure 6a, a time-dependent increase in intracellular glutamate levels was observed in EBV-positive Raji and Namalwa cells compared to the EBV-negative BJAB cells. A similar increase in intracellular glutamate levels was also observed in EBV-positive C666-1 compared to EBV-negative RPMI 2650 (Figure 6b). To confirm that the rise in glutamate levels in the infected cells was due to the activity of KGA and GAC, we treated the EBV-positive cells Raji, Namalwa, and C666-1 with 10 µM and 20 µM of BPTES, which selectively inhibits both KGA and GAC [25,26], for 72 h, and the cell lysates were analyzed for glutamate secretion. Treatment with BPTES showed a dose-dependent reduction in glutamate generation in EBV-positive Raji and Namalwa cells (Figure 6c).

Similarly, there was a significant dose-dependent reduction in the intracellular glutamate levels in BPTES-treated EBV-positive C666-1 cells than the control DMSO-treated cells (Figure 6d). These results suggest that the increased expression of KGA and GAC contributes to the higher levels of intracellular glutamate concentration in EBV-infected cells.

### 3.7. Mitochondrial GLUD1 and GLUD2 Were Upregulated and Alpha-Ketoglutarate Levels Increased in EBV-Infected Cells

**Figure 7:**
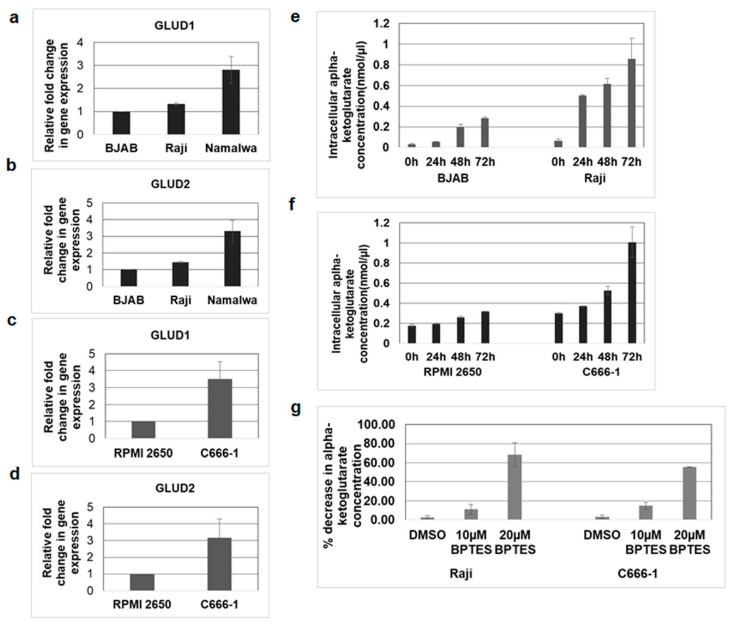
GLUD1 and GLUD2 expression and alpha-ketoglutarate levels increased in EBV-infected cells. (**a**,**b**) RNA was isolated from EBV-negative cell line BJAB and EBV-positive cell lines Raji and Namalwa. The expression of GLUD1 (**a**) and GLUD2 (**b**) was analyzed by real-time RT-PCR. (**c**,**d**) RNA isolated from EBV-negative cell line RPMI 2650 and EBV-positive cell line C666-1 were examined for GLUD1 (**c**) and GLUD2 (**d**) expression by real-time RT-PCR. The fold change in gene expression was calculated using the ∆∆CT method and normalized with respect to the expression of GAPDH. Error bars represent the mean ± SD of two independent experiments. (**e**–**g**) BJAB, Raji, RPMI 2650, and C666-1 cell lines were cultured, and alpha-ketoglutarate concentrations in cell lysates of BJAB and Raji (**e**), RPMI 2650, and C666-1 (**f**) were measured at indicated time points using alpha-ketoglutarate assay kit. The concentration of alpha-ketoglutarate is expressed in nanomoles per microliter. (**g**) Raji and C666 cell lines were treated with control DMSO or 10 µM and 20 µM BPTES. Alpha-ketoglutarate concentration was determined in the cell lysates collected after 72 h. Graphs represent the percentage decrease in alpha-ketoglutarate concentration in Raji and C666-1. Error bars represent the mean ± SD of two independent experiments.

Glutaminolysis is a series of enzyme-catalyzed reactions which starts with the conversion of glutamine into glutamate. Subsequently, glutamate is deaminated to alpha-ketoglutarate, a key molecule of the TCA cycle, by the enzymes glutamate dehydrogenase 1 (GLUD1) and GLUD2 in the mitochondria [27]. As the above findings suggested that glutamate formation from glutamine is mediated by mitochondrial KGA and GAC, we next aimed to investigate the expression of GLUD1 and GLUD2 in the infected cells. The expression of GLUD1 and GLUD2 was evaluated by real-time RT-PCR in EBV-positive Raji, Namalwa, and C666-1 as well as in the EBV-negative control BJAB and RPMI 2650 cells. We observed an increase in the expression of GLUD1 and GLUD2 in Raji and Namalwa compared to BJAB cells (Figure 7a,b). Upregulated expression of GLUD1 and GLUD2 was also observed in C666-1 cells rather than the negative control RPMI 2650 (Figure 7c,d). These findings suggest that the enzymes responsible for the production of alpha-ketoglutarate from glutamate, GLUD1 and GLUD2, were highly expressed in the EBV-infected cells.

To determine whether increased expression of GLUD1 mediates the production of alpha-ketoglutarate in EBV-infected cells, we measured the levels of alpha-ketoglutarate in Raji and C666-1 cells and their respective control cells. We found that alpha-ketoglutarate production was markedly increased in EBV-infected Raji (Figure 7e) and C666-1(Figure 7f) cells compared to their control cells. To confirm the role of mitochondrial KGA and GAC in alpha-ketoglutarate production, we blocked the conversion of glutamine to glutamate using an KGA and GAC inhibitor, BPTES [25,26]. Treatment with BPTES dose-dependently reduced the alpha-ketoglutarate levels in both Raji and C666-1 cells compared to DMSO-treated control cells (Figure 7g).

### 3.8. KGA, GAC Inhibitor, and GLUD1 Inhibitor Blocked Proliferation and Viability of EBV-Infected Cancer Cells

**Figure 8:**
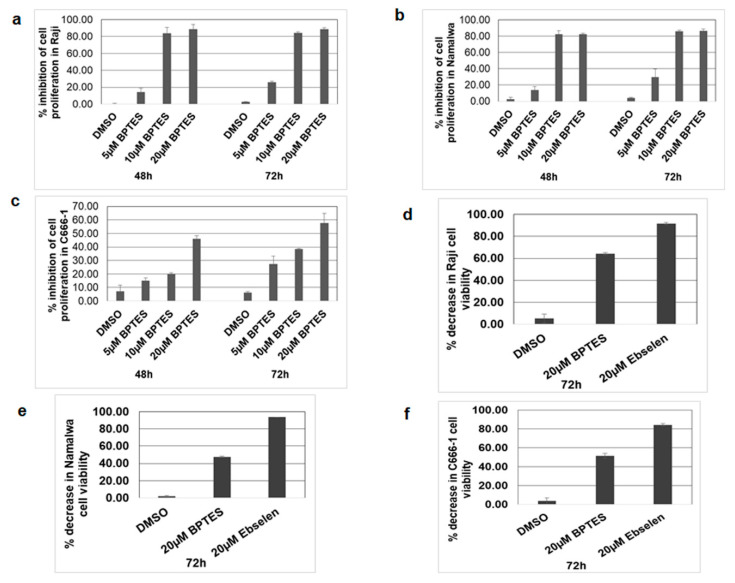
KGA, GAC, and GLUD1 inhibitor blocked cell proliferation in EBV-infected cells. (**a**–**c**) Raji, Namalwa, and C666-1 were treated with control DMSO or 5, 10, and 20 µM BPTES. After 48-h and 72-h treatments, the cells were pulsed with BrdU for 2 h and the incorporation of BrdU was measured by the BrdU cell proliferation assay kit. Graphs represent the percentage inhibition of cell proliferation in Raji (**a**), Namalwa (**b**), and C666-1 (**c**). (**d**–**f**) Raji, Namalwa, and C666-1 were treated with 20 µM BPTES and 20 µM ebselen for 72 h, after which the cell viability was monitored by MTT assay and expressed as percentage decrease in viability in Raji (**d**), Namalwa (**e**), and C666-1 (**f**). Error bars represent the mean ± SD of two independent experiments.

As we observed a significant increase in KGA and GAC, GLUD1 and GLUD2 expression, and the production of alpha-ketoglutarate in EBV-infected cells, we hypothesized that enhanced glutaminolysis through KGA and GAC may be an important component in enhancing mitochondrial metabolism and supporting cellular processes such as cell proliferation. We therefore focused on the role of KGA and GAC-mediated mitochondrial energy metabolism in the proliferation of EBV-infected cells. To investigate their role in proliferation, we treated Raji, Namalwa, and C666-1 with 10 µM and 20 µM of BPTES for 48 h and 72 h. Untreated and DMSO treated cells were used as controls. After the BPTES treatment, the cells were pulsed with BrdU and cell proliferation was determined by ELISA. Dose-dependent inhibition of cell proliferation was observed in BPTES-treated EBV-positive Raji (Figure 8a) and Namalwa cells (Figure 8b). Similarly, a significant decrease in proliferation was seen in BPTES-treated EBV-positive C666-1 cells (Figure 8c), indicating that KGA and GAC-induced glutaminolysis and mitochondrial energy metabolism contribute to the proliferation of EBV-infected cancers. To further determine the role of KGA, GAC, and GLUD1 in cell viability, we treated EBV-positive Raji, Namalwa, and C666-1 with 20 µM of BPTES for 72 h. Treatment with BPTES showed significant reductions in cell viability in Raji (Figure 8d), Namalwa (Figure 8e), and C666-1(Figure 8f). Since we observed high expression of GLUD1 in EBV-infected cells, we decided to study the effect of GLUD1 on the cell viability of EBV-infected cells, for which we treated Raji, Namalwa, and C666-1 cells with 20 µM of ebselen, which inhibits GLUD1 [28], for 72 h, along with untreated and DMSO vehicle treatments as controls. Ebselen caused an 80%–90% decrease in the viability of Raji (Figure 8d), Namalwa (Figure 8e), and C666-1 (Figure 8f) cells.

Since both KGA and GAC have similar efficiencies in catalyzing the conversion of glutamine to glutamate, our findings suggest that simultaneous expression of both the enzymes may be an adaptation to meet the high energy requirements and biosynthetic needs of rapidly growing EBV-infected cells. Treatment of EBV-infected cells with KGA and GAC inhibitor BPTES demonstrated that reduced cell proliferation and cell viability implicates GLS1 isoforms as a therapeutic intervention for EBV-associated cancers.

## 4. Discussion

EBV latent gene expression drives the oncogenic process in EBV-associated cancers. Different patterns of viral latent proteins and viral RNAs are expressed to promote the process of oncogenesis, involving the activation of oncogenic pathways, deactivation of tumor suppressors, and rewiring of metabolic pathways [7,29,30,31]. The latent genes function by activating genes which act at critical points in the metabolic pathways of the infected cells. For example, in EBV-associated lymphoma cells, the EBV membrane oncoprotein LMP1 regulates glucose metabolism by upregulating the enzymes involved in glucose metabolism, such as HK2, PDK, and PKM2, through the activation of HIF1 alpha [32]. EBV-encoded RNAs (EBERS) partly regulate the lipid metabolism and upregulation of fatty acid synthase for the synthesis of fatty acids [33]. Moreover, in EBV-associated nasopharyngeal cancers, the activation of LMP1 reprograms glutamine metabolism by increasing glutamine uptake and intracellular glutamate levels [34]. These findings provide insight into the basis of glutamine dependence in EBV-associated cancer cell metabolism. In this study, we observed that EBV upregulates the expression of two GLS1 isoforms, KGA and GAC, in EBV-positive lymphoma and nasopharyngeal cancer cells, and their localization to the mitochondria increases glutaminolysis and its end product, alpha-ketoglutarate, which may induce significant changes in the mitochondrial energy metabolism and cellular bioenergetics required for the growth and proliferation of EBV-associated cancer cells.

As both isoforms of GLS1 were equally upregulated in the EBV-induced cancers, we examined whether they are responsible for the high mitochondrial activity and cell proliferation of EBV-positive cancer cells. Overexpression of KGA and GAC leading to both cytoplasmic and mitochondrial localization is observed frequently in many types of cancer cells [18,21]. Interestingly, in EBV-associated lymphoma and nasopharyngeal cancer cells, both KGA and GAC were detected in the mitochondria, and their upregulation concomitantly elevated the intracellular glutamate levels. When we treated the cells with an inhibitor, BPTES, which inhibits both KGA and GAC, we observed a significant reduction in intracellular glutamate concentration, suggesting that both KGA and GAC have equal efficiency in the generation of glutamate [35]. Enhanced expression of KGA and GAC and glutaminolysis-dependence are important metabolic adaptations used by hepatitis C virus for cell growth [36]. In Kaposi’s sarcoma-associated herpesvirus infection, increased glutamate secretion and GLS1 expression promote cellular proliferation [37]. In addition, glutaminase has implications in the viral lifecycles of various viruses like adenovirus, herpes simplex virus 1, influenza A, and human cytomegalovirus [38]. An increased flux of mitochondrial enzymes KGA and GAC may be used by EBV-associated cancer cells to enhance glutaminolysis and support cell growth and proliferation.

There is a number of oncogenes and tumor suppressors regulating glutamine metabolism in cancer cells. Among these genes, c-Myc has long been known to regulate glutaminolysis by translationally regulating the expression of full length GLS1 in some EBV-infected cells [7]. Consistent with previous reports, our data indicate that c-Myc plays a role in the upregulation of full length KGA, as well as the shortened isoform GAC, in EBV-positive cells for the enhancement of glutaminolysis. Inhibition of c-Myc concurrently decreased the expression of both KGA and GAC in the infected cells, and their downregulation in turn decreased the intracellular glutamate level and the proliferation of the infected cells. A significant inhibition of KGA and GAC proteins in c-Myc knockdown and c-Myc inhibitor treated cells supports our concept that c-Myc contributes to the KGA and GAC-dependent activation of glutaminolysis, which may result in energy production and the rapid proliferation of the infected cells.

Our further analysis revealed that an increased expression of GLUD1 and GLUD2 that converts mitochondrial glutamate to alpha-ketoglutarate elevated the production of alpha-ketoglutarate in the infected cells. The outcome of enhanced production of alpha ketoglutarate is to replenish the TCA cycle to generate ATP and other intermediate metabolites of biosynthetic pathway. The elevated levels of alpha-ketoglutarate in EBV-infected cells were reduced upon the inhibition of KGA and GAC, making them essential molecules in mediating the mitochondrial energy metabolism via glutaminolysis, which could support the proliferation of the infected cells. In the infected cells, these enzymes may be regulated by multiple latent genes. Our studies show that higher levels of KGA, GAC, GLUD1, and GLUD2 expression occur in latency type 3 Namalwa cells than the latency type 2 Raji cells, indicating a close relationship between the patterns of latent gene expression involved in the mitochondrial metabolic pathways. However, future studies are needed to understand how EBV genes leads to the activation of these mitochondrial genes and the molecular basis of their functional link with the mitochondrial metabolic network.

We further examined the functionality of KGA and GAC activity in the proliferation and viability of EBV-infected cells. BPTES, an allosteric inhibitor of KGA and GAC, has been shown to reduce proliferation in a few cancer types [39,40]. Our data demonstrated that KGA and GAC inhibition using BPTES not only inhibited the proliferation of the infected cells but also induced cell death. Additionally, inhibition of GLUD1 using ebselen resulted in greater cell death than individual KGA and GAC-mediated inhibition with BPTES. However, studies have also reported that ebselen inhibits GLS1 [41] and thus the combined inhibition of GLS1 and GLUD1 upon treatment with ebselen may be accounted for by a greater reduction in the viability of EBV-infected cells.

Taken together, we provide evidence that the enhanced expression of mitochondrial GLS1 isoforms plays a crucial role in initiating and maintaining the glutaminolytic process to drive the production of energy by the TCA cycle in EBV-infected cells. These findings open up possibilities for targeting KGA, GAC, and GLUD1 inhibitors as a therapeutic strategy for the treatment of EBV-associated cancers. The mitochondrial GLS1 is a potential therapeutic target in many cancer cells [42,43,44]. Several GLS1 inhibitors have been shown to have potent anti-tumor efficiencies and are effective in controlling the proliferation of cancer cells. CB-839, a GLS1 inhibitor, is currently in its phase I clinical trial for the treatment of various cancers. Therefore, the present study suggests a promising role of the inhibitors of GLS1 and its isoforms for the treatment of EBV-associated cancers.

## Figures and Tables

**Table 1:** List of antibodies.

Sl No.	Antibody	Make	Catalog No.
1	Rabbit anti-glutaminase antibody	Thermo Fisher Scientific	710997
2	Rabbit anti- KGA/GAC antibody	Proteintech	12855-1-AP
3	Rabbit anti-β-actin antibody	Abcam	ab8227
4	Mouse anti-c-Myc (9E10) antibody	Santa Cruz	sc-40
6	Rabbit anti-VDAC antibody	Millipore	AB10527
7	Anti-mouse secondary antibody linked to HRP	Thermo Fisher Scientific	31430
8	Anti-rabbit secondary antibody linked to HRP	Thermo Fisher Scientific	31460
9	Anti-rabbit secondary antibody conjugated to Alexa Flour 594	Thermo Fisher Scientific	A11012
10	Anti-rabbit secondary antibody conjugated to Alexa Flour 488	Thermo Fisher Scientific	A11034

**Table 2:** Primer sequences used for real-time PCR amplifications.

Gene	Forward primer 5′-3′	Reverse primer 5′-3′
KGA	CTGGAAGCCTGCAAAGTAAAC	TGAGGTGTGTACTGGACTTGG
GAC	CCTCGAAGAGAAGGTGGTGATC	TGTCCTCATTTGACTCAGGTGAC
GLS1	GTCACGATCTTGTTTCTCTGTG	GTCCAAAGAGCAGTGCTTCATCCATG
GLUD1	GACACCAGGGTTTGGAGATAAA	TCAGACTCACCAACAGCAATAC
GLUD2	ATCGGGTGCATCTGAGAAAG	CAGGTCCAATCCCAGGTTATAC
GAPDH	AGGGCTGCTTTTAACTCTGGT	CCCCACTTGATTTTGGAGGGA
